# Cutaneous papillomaviruses infection rewires fatty-acid metabolism

**DOI:** 10.3389/fcimb.2026.1860417

**Published:** 2026-08-19

**Authors:** Xiaoyan Liu, Dan Li, Fangzhou Cai, Yan Li, Wei Wang, Junhua Liao, Shijun Shan

**Affiliations:** 1 Department of Dermatology, Xiang’an Hospital of Xiamen University, School of Medicine, Xiamen University, Xiamen, Fujian, China; 2 National Center of Technology Innovation for animal model. State Key Laboratory of Respiratory Health and Multimorbidity. Key Laboratory of Pathogen Infection Prevention and Control (Peking Union Medical College), Ministry of Education. NHC Key Laboratory of Comparative Medicine. Institute of Laboratory Animal Science, CAMS & PUMC, Beijing, China; 3 Department of Rheumatology and Clinical Immunology, the First Affiliated Hospital of Xiamen University, School of Medicine, Xiamen University, Xiamen, Fujian, China; 4 Universite Paris Dauphine, Paris, France; 5 College of Mathematical Medicine, Zhejiang Normal University, Jinhua, Zhejiang, China

**Keywords:** cutaneous warts, fatty-acid metabolism, immune cell infiltration, papillomavirus infection, transcriptome analysis

## Abstract

**Background:**

Cutaneous warts are common HPV-induced lesions with limited treatments for recalcitrant cases. Fatty-acid metabolism (FAM) maintains epidermal homeostasis, but whether papillomavirus infection actively suppresses FAM programs and whether restoring FAM-related regulators can modulate keratinocyte dysfunction remain unclear.

**Methods:**

We established a murine MmuPV1 wart model and performed bulk RNA-seq. FAM-related DEGs were identified via network analysis and machine learning. Immune infiltration was assessed by CIBERSORT. *In vitro* validation used HPV16 E6/E7-transfected HaCaT cells.

**Results:**

Transcriptome analysis revealed significant suppression of FAM pathways in wart lesions. Four key FAM-related differentially expressed genes (Nr1h3, Dgat1, Scd1, and Fabp4) were identified from RNA-seq-based bioinformatic analyses and further supported by *in vitro* E6/E7-related expression assays. *In vitro*, HPV16 E6/E7 expression reduced these genes in HaCaT keratinocytes. NR1H3 overexpression partially attenuated E6/E7-induced proliferation and differentiation abnormalities, lipid-droplet accumulation, and IL-6/CCL2 secretion, with partial restoration of ABCA1 expression and inhibition of NF-kB activation.

**Conclusions:**

Papillomavirus infection is associated with coordinated suppression of FAM-related programs. NR1H3, Dgat1, Scd1, and Fabp4 represent candidate metabolic regulatory nodes; their therapeutic relevance requires further *in vivo* validation.

## Graphical Abstract

**Figure:**
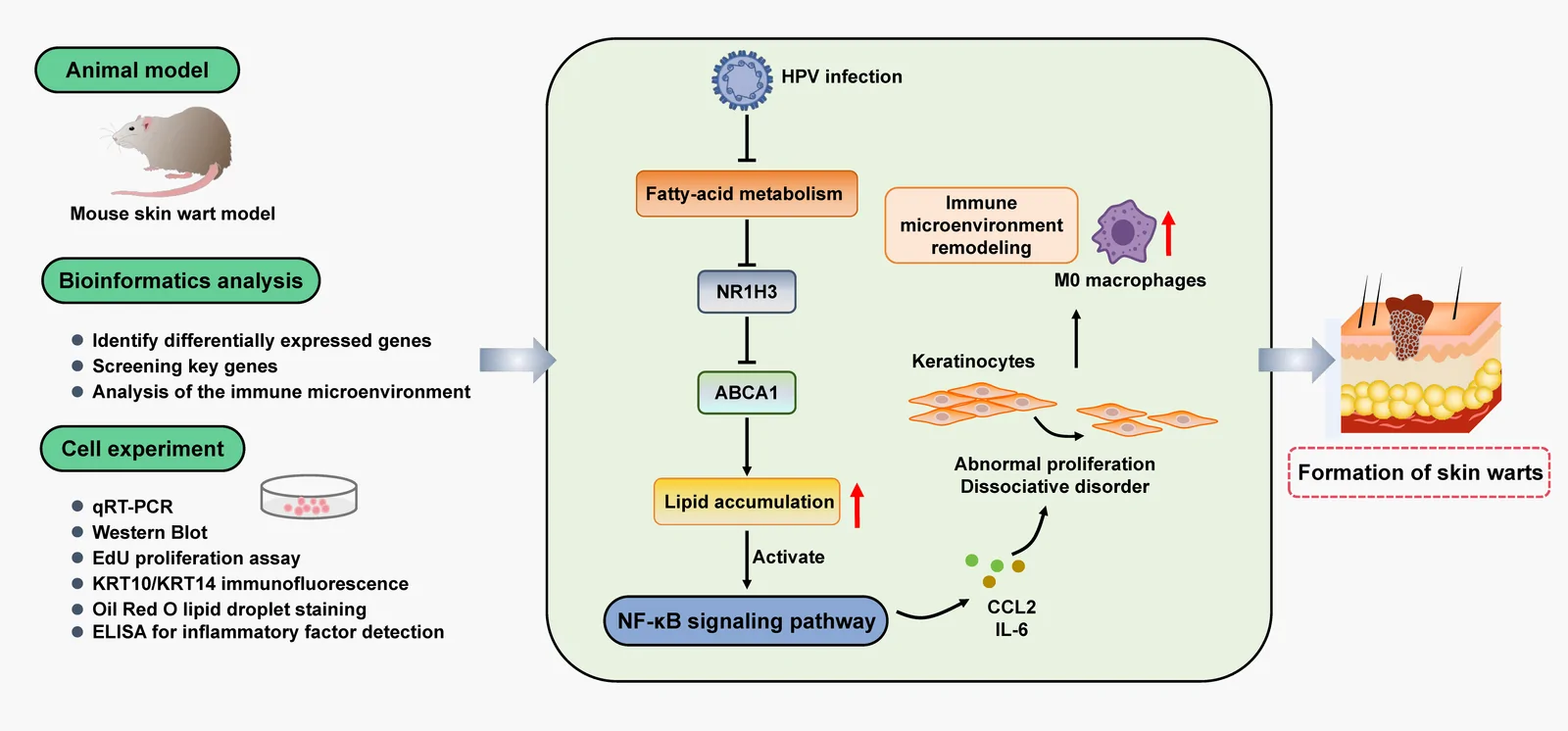
Papillomavirus infection is associated with fatty-acid metabolic remodeling and NR1H3/ABCA1-related inflammatory signaling in cutaneous wart formation.

## Introduction

Cutaneous warts, most commonly caused by human papillomavirus (HPV) types 1, 2, 4, and 7, are frequent benign skin lesions that typically present as hyperkeratotic, cauliflower-like verrucous papules (Mesgarzadeh et al., 2022). Children and adolescents are disproportionately affected because of immature immune responses and frequent close-contact activities; prevalence in these age groups can reach up to 10% (Zhu et al., 2022). Approximately 35% of warts are recalcitrant, showing no response to therapy or relapsing soon after treatment, which leads to substantial physical and psychological morbidity and makes clinical management particularly challenging (Tantawy et al., 2022; Elyamany et al., 2025).

Fatty acids in the skin serve as energy substrates and membrane-lipid building blocks, activate receptors, are converted into lipid mediators, and drive the biosynthesis of complex lipids; their cutaneous content and composition strongly influence skin structure and function (Lin and Khnykin, 2014; Yang et al., 2020). Dysregulation of fatty-acid metabolism (FAM) has also been closely linked to epithelial barrier impairment, altered cytokine signaling, and chronic inflammatory skin disease, suggesting that lipid-metabolic rewiring may be a biologically relevant component of papillomavirus-induced lesions.

Lipid droplets are dynamic endoplasmic-reticulum-derived organelles that store neutral lipids, buffer lipotoxic stress, and provide lipid substrates for signaling mediators. In inflammatory and infectious settings, lipid droplets can act as hubs for eicosanoid generation and cytokine-linked metabolic remodeling, thereby influencing immune-cell recruitment and local inflammatory tone (Bozza and Viola, 2010; Olzmann and Carvalho, 2019). This biology provides a mechanistic bridge between the FAM signatures observed in wart tissue and the lipid-droplet and cytokine phenotypes tested in our keratinocyte experiments.

Here, we asked whether papillomavirus-induced cutaneous wart formation is associated with coordinated FAM remodeling, and whether early viral oncogene activity can suppress candidate FAM regulators in keratinocytes. We used bulk RNA-seq to identify FAM-related differentially expressed genes (FAM-DEGs) in MmuPV1-induced wart lesions, constructed a transcription-factor regulatory network centered on hub FAM-DEGs, and characterized relationships between these genes and inferred immune-cell infiltration. We then used HPV16 E6/E7-transfected HaCaT cells to test whether viral early-gene expression downregulates the same candidate FAM nodes and whether restoring NR1H3 can modulate keratinocyte proliferation, differentiation, lipid accumulation, and inflammatory signaling. These analyses were designed to establish association and mechanistic plausibility, while definitive therapeutic validation will require *in vivo* perturbation studies.

## Materials and methods

### MmuPV1 tail-skin infection model and experimental design

Female NU-Foxn1^nu mice (6-8 weeks, 18–25 g) were obtained from Beijing Vital River Laboratory Animal Technology Co., Ltd. and housed in an ABSL-2 facility at the Institute of Laboratory Animal Sciences, Chinese Academy of Medical Sciences (20-26 deg C, 40-70% humidity, 12 h/12 h light-dark cycle; food and water ad libitum). We used a previously described MmuPV1 tail-skin infection approach (Hu et al., 2017; Li et al., 2021) rather than establishing a new viral model *de novo*. Mice were randomized to model or normal groups. For infection, model mice were anesthetized with isoflurane; tail skin was lightly scarified (20 strokes with a sterile blade). The MmuPV1 viral stock was kindly provided by Jiafen Hu and was prepared as a laboratory inoculum. For infection, 10 μL of viral inoculum containing 1.5 × 10^8^ viral DNA copies was applied to the site and air-dried before mice were returned to cages. Animals were inspected weekly; by day 30 post-infection, approximately 50% exhibited lesions. Only mice that developed visible warts were included in the final model group for RNA-seq. The laboratory mice that did not show any visible warty lesions by the 60th day after infection were excluded from the RNA-seq analysis. Wart volumes were measured on days 30, 45, and 60, and tissues from both groups were collected on day 60. Caliper-based volumes were calculated using an ellipsoidal model: V = pi/6 x length x width^2. At the end stage of the experiment, euthanasia was carried out by intraperitoneal injection of an excessive dose of pentobarbital sodium (100 mg/kg). All animal experiments were approved by the Institutional Animal Care and Use Committee (IACUC) of the Institute of Laboratory Animal Sciences, Chinese Academy of Medical Sciences (Approval No. WW23002). All methods were performed in accordance with the relevant guidelines and regulations, including the National Institutes of Health Guide for the Care and Use of Laboratory Animals. This study is reported in accordance with the ARRIVE guidelines.

### Detection of MmuPV1 E2 DNA by PCR and agarose gel electrophoresis

Total DNA was extracted from skin tissues of the MmuPV1-infected model and uninfected control groups using the DNeasy Blood & Tissue Kit (QIAGEN; 69506) according to the manufacturer’s instructions. The MmuPV1 E2 region was amplified using primers MmuPV1_E2_1 (5′-GCCCGAAGACAACACCGCCACG-3′) and MmuPV1_E2_2 (5′-CCTCCGCCTCGTCCCCAAAAAATGG-3′). Each 25-μL PCR mixture contained 2.5 μL of 10× High Fidelity PCR Buffer, 0.5 μL of 10 mmol/L dNTP Mix, 1 μL of 50 mmol/L MgSO_4_, 1 μL of each primer, 3 μL of tissue DNA, 0.1 μL of Platinum Taq DNA Polymerase High Fidelity, and 15.9 μL of nuclease-free water. Amplification was performed at 94 °C for 10 min, followed by 35 cycles of 94 °C for 15 s, 60 °C for 30 s, and 68 °C for 45 s, with a final extension at 68 °C for 10 min. PCR products were resolved by agarose gel electrophoresis using a DL2000 DNA Marker (TaKaRa; 3427A) and visualized with a gel documentation system. Bands of the expected MmuPV1 E2 amplicon size were recorded. MmuPV1-positive DNA, DNA extracted from uninfected tissue, and a no-template control were included as the positive control, biological negative control, and no-template control, respectively.

Viral load was quantified by qPCR using a standard curve generated from serially diluted pMusPV plasmid of known copy numbers. The MmuPV1 E2 gene was amplified using the same primers as conventional PCR and a FAM-TGCCCTTTCAGTGGGTTGAGGACAG-MGB probe. Each 20-μL reaction contained 10 μL of 2× QuantiTect Probe PCR Master Mix, 1 μL each of forward and reverse primers, 0.5 μL of probe, 1 μL of template DNA, and 6.5 μL of RNase-free water. Cycling conditions were 50 °C for 2 min, 95 °C for 10 min, followed by 40 cycles of 95 °C for 15 s and 60 °C for 1 min.

### H&E staining

Tail-skin tissues, including the central wart lesion and adjacent epithelium in the model group or matched tail skin in the normal group, were fixed, dehydrated, embedded in paraffin, and sectioned at 4 um. Sections were deparaffinized in xylene and rehydrated through graded ethanol (100%, 95%, 85%, 75%) prior to staining. Sections were stained with hematoxylin (H&E kit; Solarbio, Beijing, China; G1120), rinsed, differentiated for 1 min, washed in running tap water, counterstained with eosin, then rapidly dehydrated, cleared, and mounted with neutral balsam (Solarbio; G8590). At least three non-overlapping representative fields per section were examined using a light microscope (Lattice SIM 3, ZEISS), and histopathological features including epidermal hyperplasia, rete-ridge extension, vacuolated keratinocytes, and inflammatory-cell infiltration were recorded.

### Bulk RNA-seq data processing and DEG analysis

Total RNA extraction was performed as described in the RNA extraction section. RNA-seq libraries were constructed using the TruSeq Stranded Total RNA Library Prep Kit (RS-122-2201, Illumina), and paired-end sequencing was performed on an Illumina NovaSeq 6000 platform. Clean reads were generated after standard QC (removal of low-quality and adaptor-contaminated reads; correction of GC bias) and aligned to the mouse genome (mm10) using HISAT2. Reads with MAPQ < 10, multimapped reads, and unpaired reads were excluded. PCA and standardized boxplots were computed from FPKM values for exploratory QC. Differential expression was performed in DESeq2 using raw count matrices; two-sided Wald tests were applied and P values were adjusted by the Benjamini-Hochberg method. DEGs were defined as genes with FDR < 0.05 and |log2FC| > 1. Volcano plots, heatmaps, and pathway enrichment analyses were generated using these thresholds.

### Functional enrichment analysis

Gene set enrichment analysis (GSEA) was performed with gseGO (clusterProfiler) using org.Mm.eg.db for annotation and a signed, preranked gene list as input. Parameters were minGSSize = 10, maxGSSize = 500, pAdjustMethod = “BH”, and nPermSimple = 10000. Significance was defined as |NES| > 1 with Pvalue < 0.05. Pathway enrichment analysis of DEGs for GO and KEGG pathways was conducted with enrich GO and enrich KEGG (threshold P < 0.05). Plots were generated with ggplot2.

### Identification and functional enrichment analysis of FAM-DEGs

To assemble a FAM-related gene list, we used GO.db and org.Mm.eg.db to define genes annotated to fatty acid metabolic process (GO:0006631) and all of its BP offspring (via GOBPOFFSPRING). Intersecting these genes with DEGs yielded the FAM-DEGs, whose expression was visualized as heatmaps in ggplot2. Pairwise correlations among FAM-DEGs were computed and plotted with corrplot; correlation coefficients indicate signed Pearson r values, color encodes direction and magnitude, and asterisks indicate the corresponding two-sided P-value levels. Functional enrichment (GO and KEGG) of FAM-DEGs was performed using ClueGO in Cytoscape.

### Identification of hub FAM-DEGs

Protein–protein interaction (PPI) among the 57 FAM-DEGs were queried in STRING. Candidate hubs were prioritized using cytoHubba (Cytoscape) with the Maximal Clique Centrality (MCC) metric and, in parallel, by a random-forest (RF) classifier that uses bootstrap aggregation of decision trees. The core FAM-DEGs were defined as the intersection of the top 20 MCC-ranked genes and the RF-selected genes (n = 4). Expression of core FAM-DEGs across groups was visualized with boxplots.

### Construction of the regulatory network for hub FAM-DEGs

Upstream regulators of hub FAM-DEGs were inferred in Cytoscape using iRegulon, which integrates *de novo* motif discovery with cis-regulatory track enrichment to recover TF–target regulons. Stringent enrichment thresholds were applied, restricting searches to proximal and distal regulatory regions. Predicted TF–target pairs were assembled into a FAM-DEG-TF network and visualized in Cytoscape. Node degree and centrality metrics were calculated to highlight putative key regulators.

### Immune cell deconvolution and gene–immune correlation analysis

Immune composition was inferred from bulk RNA-seq (FPKM) using CIBERSORT with the LM22 leukocyte signature. Mouse gene symbols were mapped one-to-one to human orthologs (homoloGene/biomaRt), duplicates were removed, and the human-symbol matrix was analyzed with CIBERSORT.R. Samples (model M1–M4; control N1–N3) yielded estimates for 22 leukocyte subsets, visualized as stacked bars and boxplots; between-group differences were assessed by two-sided Wilcoxon tests. Expression of core FAM-DEGs was combined with CIBERSORT fractions to compute pairwise Pearson correlations on the transposed matrix; two-sided P values were obtained with cor.mtest (95% CI; no multiple-testing correction). Gene–immune correlations were extracted for the predefined gene list and plotted as a ggplot2 heatmap, with significance indicated by asterisk symbols according to the corresponding P values.

### Cell culture and transfection

The human keratinocyte cell line HaCaT was maintained in Dulbecco’s Modified Eagle Medium (DMEM) supplemented with 10% fetal bovine serum (FBS) and 1% penicillin-streptomycin at 37 deg C in a 5% CO2 atmosphere. For transient transfection experiments, cells were seeded in 6-well plates or appropriate culture vessels and transfected at approximately 70-80% confluence using Lipofectamine 3000 (Invitrogen, USA) according to the manufacturer’s instructions. HPV16 E6/E7 co-expression plasmids, NR1H3 overexpression plasmid, and their corresponding empty vector or negative-control plasmids were used as indicated; OE-NC denotes the empty/negative-control overexpression vector. Cells were transfected with 2 μg total plasmid DNA per well of a six-well plate, balanced with empty vector where necessary. After 6 h of transfection, the medium was replaced with fresh complete medium. Cells were harvested at 24 h for qRT-PCR and 48 h for protein and functional assays.

### RNA extraction and quantitative real-time PCR

Total RNA was extracted using TRIzol reagent (Invitrogen, 15596018CN) and reverse transcribed into cDNA using PrimeScript RT kit (Takara, Japan, RR037A). Real-time quantitative PCR (qRT-PCR) analysis was performed using the SYBR Green chemistry kit (Q713-02, Vazyme)on a real-time fluorescence quantitative PCR detection system (Thermo Fisher Scientific, QuantStudio™ 5). The primer sequences are shown in **Table 1**. The relative expression levels were calculated using the 2^−ΔΔCt^ method. Each experiment was repeated three times.

**Table 1:** Primer sequences.

Gene	Primer sequences (5’-3’)
E6	F-TTACCACAGTTATGCACAGAGC
	R-TTTTCTTCAGGACACAGTGGC
E7	F-TAAATGACAGCTCAGAGGAGG
	R-CCCATCTGTTCTCAGAAACCATAA
NR1H3	F-TCCTGTCAGAAGAACAGATCC
	R-TCGGCATCATTGAGTTGCAG
DGAT1	F-CTTCTTCTACTGGCTCTTCC
	R-TTGAGCACGTAGTAGTCGTG
SCD1	F-TTGCCAACACAATGGCATTCC
	R-ATAAGGACGATATCCGAAGAGG
FABP4	F-GGTACCATGGAAACTTGTCTC
	R-AGGTTATGGTGCTCTTGACTTTCC
GAPDH	F-AAGATCATCAGCAATGCCTCC
	R-AGGTTTTTCTAGACGGCAGG

### Western blot analysis

Cells were lysed in RIPA buffer containing protease and phosphatase inhibitors (Thermo Fisher Scientific, A32959). Protein concentrations were determined using a BCA assay kit (Thermo Fisher Scientific, A55865). Equal amounts of protein (20-30 ug) were separated by SDS-PAGE and transferred onto PVDF membranes. After blocking with 5% non-fat milk, membranes were incubated overnight at 4 deg C with primary antibodies against NR1H3 (Proteintech, 14351-1-AP, 1:10000, Rabbit), DGAT1 (Abcam, ab54037, 1:500, Rabbit), SCD1 (Abcam, ab236868, 1:1000, Rabbit), FABP4 (Abcam, ab92501, 1:5000, Rabbit), ABCA1 (Proteintech, 26564-1-AP, 1:1000, Rabbit), p-p65 (CST, #3033, 1:1000, Rabbit), IκBα (AFFINITY, AF5002, 1:2000, Rabbit), and p-IκBα (Abcam, ab133462, 1:10000, Rabbit), followed by HRP-conjugated goat anti-rabbit IgG secondary antibody (BIOSS, bs-0295G-HRP, 1:3000). The listed primary antibodies were used with the appropriate HRP-conjugated secondary antibody according to the host species. Protein bands were visualized using an ECL detection system (Tanon) and quantified with ImageJ software. GAPDH (Proteintech, 81640-5-RR, 1:10000, Rabbit) or β-actin (Proteintech, 81115-1-RR, 1:10000, Rabbit) served as loading controls.

### 5-Ethynyl-2’-deoxyuridine proliferation assay

For EdU proliferation assays, HaCaT cells were assigned to the same transfection groups used for the rescue experiments: control, vector control, E6/E7 + OE-NC, NR1H3 overexpression, and E6/E7 + NR1H3 overexpression, as applicable. Cell proliferation was evaluated using the EdU cell proliferation assay kit (Beyotime, China, C0071S), following the manufacturer’s instructions. Transfected cells were incubated with 10 uM EdU for 2 h, fixed with 4% paraformaldehyde, and stained. Cell nuclei were re-stained with DAPI. EdU-positive cells were observed under a fluorescence microscope (Olympus IX73), and the number of EdU-positive cells in three randomly selected fields of view in each well was quantified using ImageJ software.

### Immunofluorescence staining

For immunofluorescence staining, cells were cultured on coverslips to approximately 60-70% confluence after transfection, fixed with 4% paraformaldehyde for 20 min, permeabilized with 0.1% Triton X-100 for 10 min, and blocked with 5% bovine serum albumin (BSA) for 30 min. The cells were incubated overnight at 4 deg C with primary antibodies against KRT14 (ET1610-42, HUABIO, 1:500) and KRT10 (ET1609-75, HUABIO, 1:200), followed by incubation with fluorescently labeled secondary antibodies iFluor™ 488 (HA1121, HUABIO, 1:1000) 50min. The cell nuclei were stained with DAPI 10min. Fluorescence images were captured using a fluorescence microscope, and the relative fluorescence intensity was quantified using ImageJ software.

### Enzyme-linked immunosorbent assay

After transfection for 48 h, the culture supernatant was collected and centrifuged to remove cell debris. The levels of IL-6 and CCL2 were detected using commercial enzyme-linked immunosorbent assay kits (Abcam) according to the manufacturer’s instructions. Color development was performed using TMB substrate solution supplied with the ELISA kit, and the reaction was stopped according to the manufacturer’s instructions before absorbance was read at 450 nm (BioTek).

### Oil red O staining

The accumulation of lipid droplets was evaluated by Oil Red O staining. Cells were fixed with 4% paraformaldehyde for 10–15 min at room temperature, washed twice with PBS, and incubated with 60% isopropanol for 1–2 min. Cells were incubated with Oil Red O working solution (Sigma-Aldrich, O1391; 0.5% in isopropanol) at room temperature in the dark for 10–20 min. Cells were differentiated with 60% isopropanol for 5-10 s, and rinsed with distilled water until no excess stain was visible, and imaged under an optical microscope. Oil Red O-positive areas were quantified using ImageJ software and expressed as a percentage of the total cell area.

### Statistical analysis

Analyses were conducted in R (v4.4.1) and GraphPad Prism (v10.0.0). Normality was evaluated by the Shapiro–Wilk test. For independent two-group comparisons, two-tailed unpaired t tests were used when normality was not rejected (Welch’s correction for unequal variances) and the Mann–Whitney U test otherwise. Exact two-sided P values and 95% CIs are reported (α = 0.05). The data of wart volume did not follow a normal distribution. The results were presented in the form of median and interquartile range. Longitudinal, paired wart-volume data (30, 45, 60 days) were analyzed with the Friedman test followed by Dunn’s multiple comparisons (adjusted P values). The *in vitro* experimental data are presented in the form of mean ± standard deviation (SD), and the data come from at least three independent experiments. The differences between groups are analyzed by one-way analysis of variance (ANOVA), and *post-hoc* tests using Tukey’s method are conducted with the software Prism 10.0. A p-value < 0.05 is considered statistically significant.

## Results

### MmuPV1-infected mouse model

**Figure 1:**
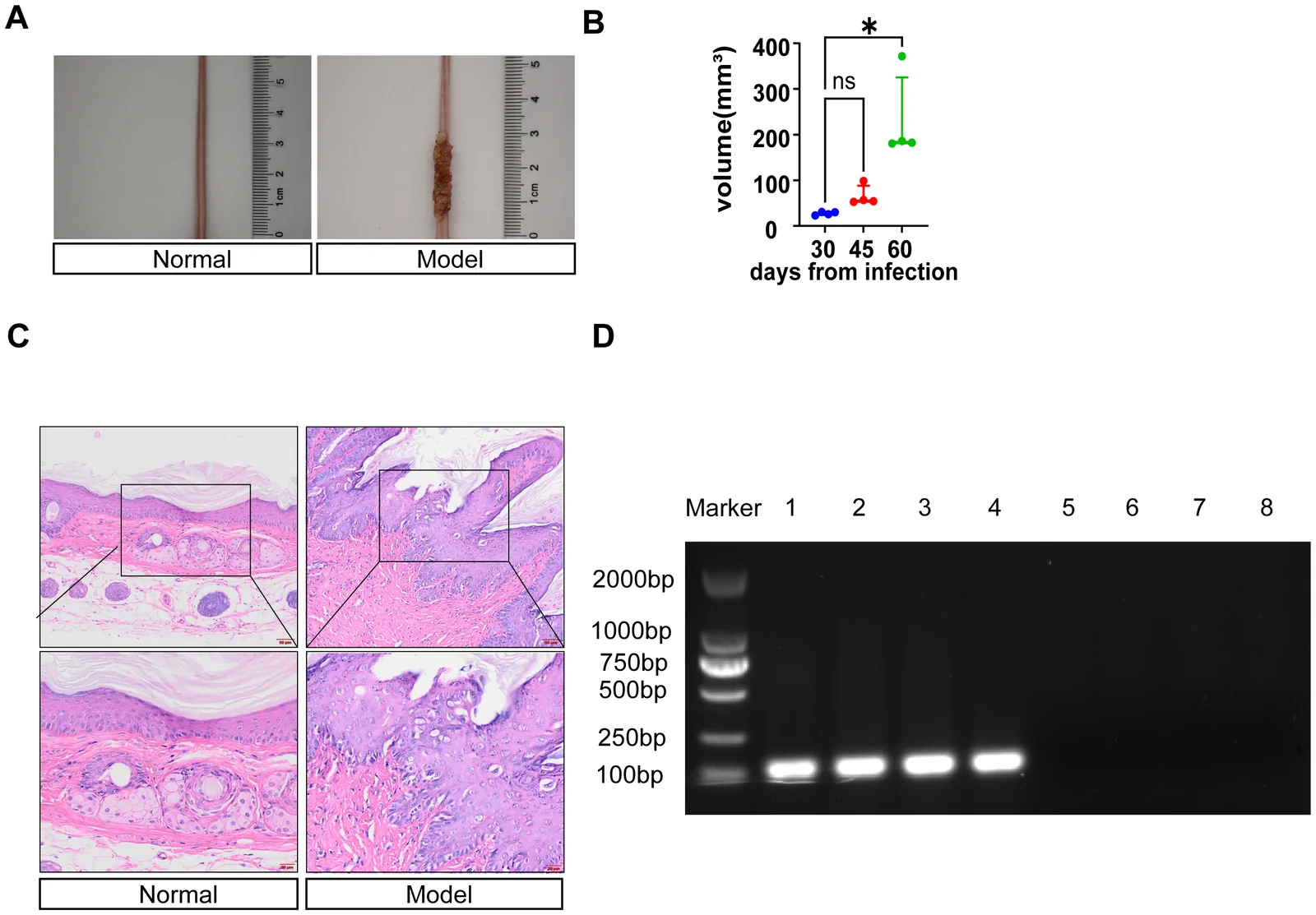
Establishment and validation of the MmuPV1-induced tail wart model in mice. **(A)** Representative tail-skin images from normal and MmuPV1-infected groups (Normal group: n = 3; Model group: n = 4). **(B)** Dot plot of wart volume in the infected group at 30, 45, and 60 days post-infection (longitudinal, paired). ns, P > 0.05; *P < 0.05 vs. 30 days. The data are presented as the median and interquartile range. **(C)** H&E-stained sections showing histopathological features across groups. Scale bars: 50 μm (upper panels), 20 μm (lower panels). **(D)** MmuPV1 DNA within the E2 region was detected by PCR in tail skin tissues from MmuPV1-infected mice and uninfected control mice. Lanes 1-3, DNA extracted from the tail skin tissues of MmuPV1-infected mice; lane 4, pMusPV plasmid as the positive control; lanes 5-7, DNA extracted from the tail skin tissues of uninfected control mice; lane 8, nuclease-free water as the no-template control.

We established a murine cutaneous wart model by lightly scarifying the tail skin of NU-Foxn1nu mice, followed by topical inoculation with MmuPV1. Visible wart lesions appeared at approximately day 30 post-inoculation, and tissues were harvested on day 60 for analysis and photo documentation (**Figure 1A**). Wart volumes were measured on days 30, 45, and 60 (**Figure 1B**). In the MmuPV1-infected group, the epidermis was markedly thickened with downward extension of rete ridges, and numerous vacuolated keratinocytes, typical of common warts, were observed (**Figure 1C**). To verify the presence of MmuPV1 DNA in the infected skin tissues, total DNA was extracted from the skin tissues of infected and uninfected control mice and subjected to PCR amplification using MmuPV1 E2-specific primers. Agarose gel electrophoresis revealed a specific 97-bp amplicon in all samples from the infected group, whereas no corresponding band was detected in the uninfected control group, confirming the presence of MmuPV1 DNA in the infected skin tissues (**Figure 1D**). The viral loads in mouse tail tissues from each group are shown in **Supplementary Figure 1**.

### Sample-level quality control and concordance assessment

**Figure 2:**
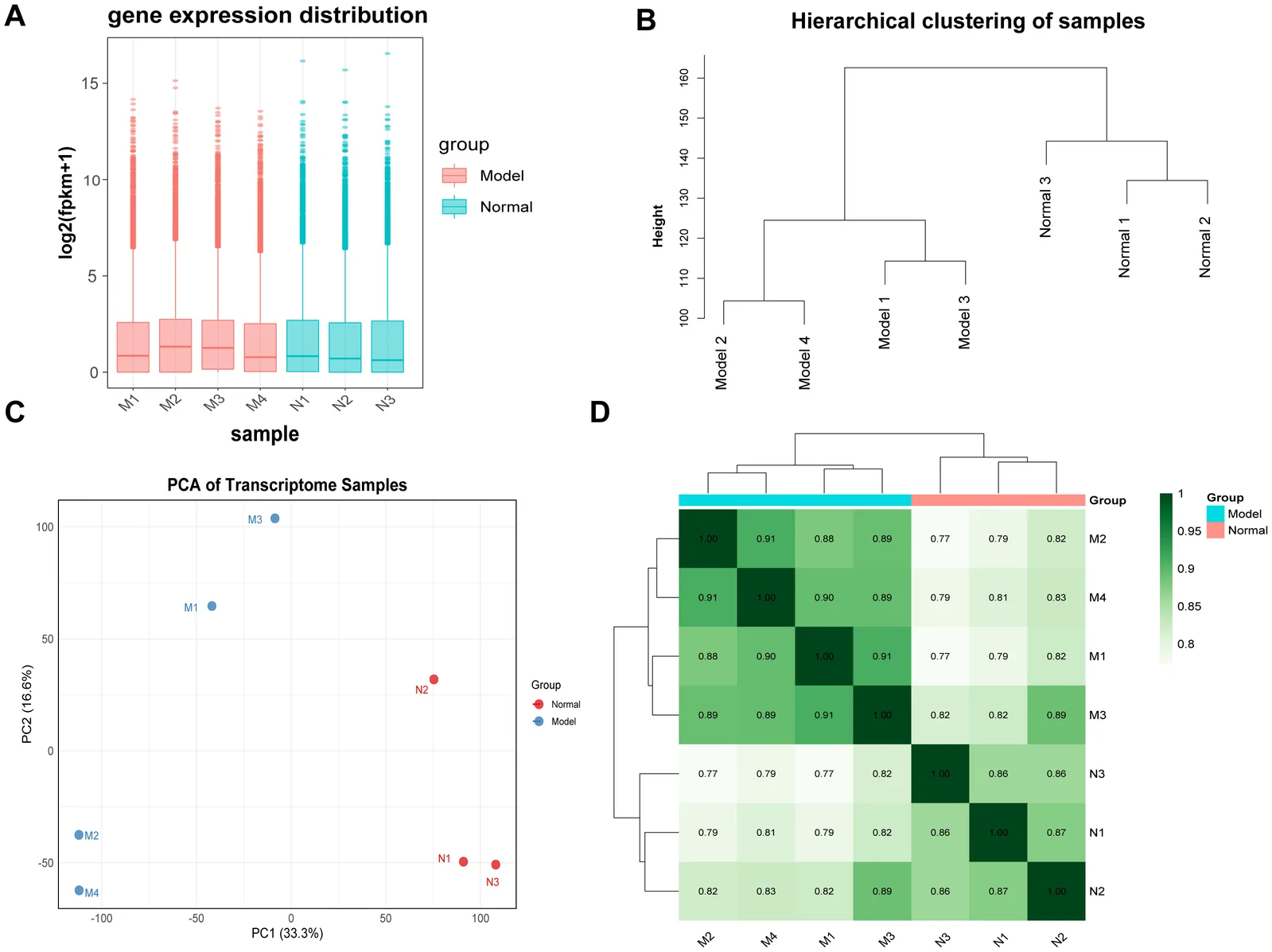
Sample normalization and clustering. **(A)** Boxplots of log2(FPKM + 1)–normalized expression across samples. **(B)** Hierarchical clustering of samples based on whole-transcriptome expression profiles. **(C)** Principal component analysis of whole-transcriptome profiles (variance explained by PC1 and PC2 indicated). **(D)** Sample-to-sample Pearson correlation heatmap with hierarchical clustering.

Bulk RNA-seq quality control and exploratory analyses are summarized in **Figure 2**. Transcript counts from seven samples (M1–M4, N1–N3) were transformed as log2(FPKM + 1) for box plots; medians, interquartile ranges, and whiskers were highly consistent across samples, indicating no evident batch effects or quality anomalies (**Figure 2A**). Hierarchical clustering of whole-transcriptome profiles separated the seven samples into two distinct branches, corresponding to the model (M1–M4) and normal (N1–N3) groups, with between-group distances exceeding within-group distances (**Figure 2B**). Principal component analysis showed that PC1 and PC2 explained 33.3% and 16.6% of the variance, respectively; samples segregated prominently along PC1, with the model group (blue) on the negative axis and the normal group (red) on the positive axis, indicating disease status as the primary driver of variance (**Figure 2C**). A correlation heatmap with hierarchical clustering based on Pearson coefficients likewise demonstrated clear separation between groups (**Figure 2D**).

### DEGs and functional enrichment in the cutaneous wart model versus normal skin

**Figure 3:**
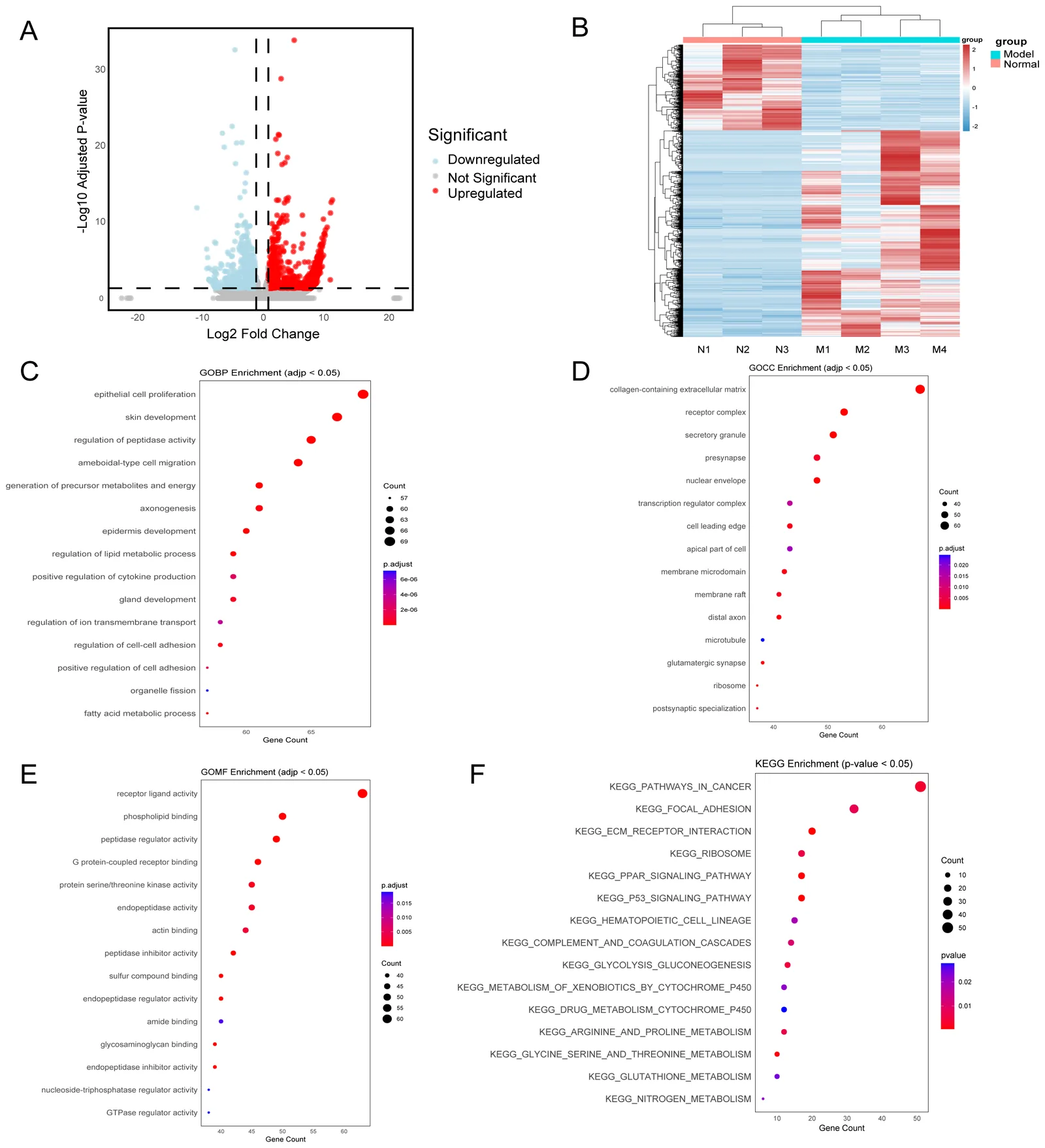
Differential expression and pathway enrichment analyses. **(A)** Volcano plot of DEGs (log2FC vs −log10[FDR]); red indicates up-regulated genes, while blue represents down-regulated genes. **(B)** Heatmap of DEGs across samples (row-scaled expression; higher, red; lower, blue). **(C–F)** Pathway enrichment analysis: **(C)** GO Biological Process (GOBP), **(D)** GO Cellular Component (GOCC), **(E)** GO Molecular Function (GOMF), and **(F)** KEGG pathways. KEGG pathway maps were generated using the KEGG database (Kanehisa et al., 2025).

**Figure 4:**
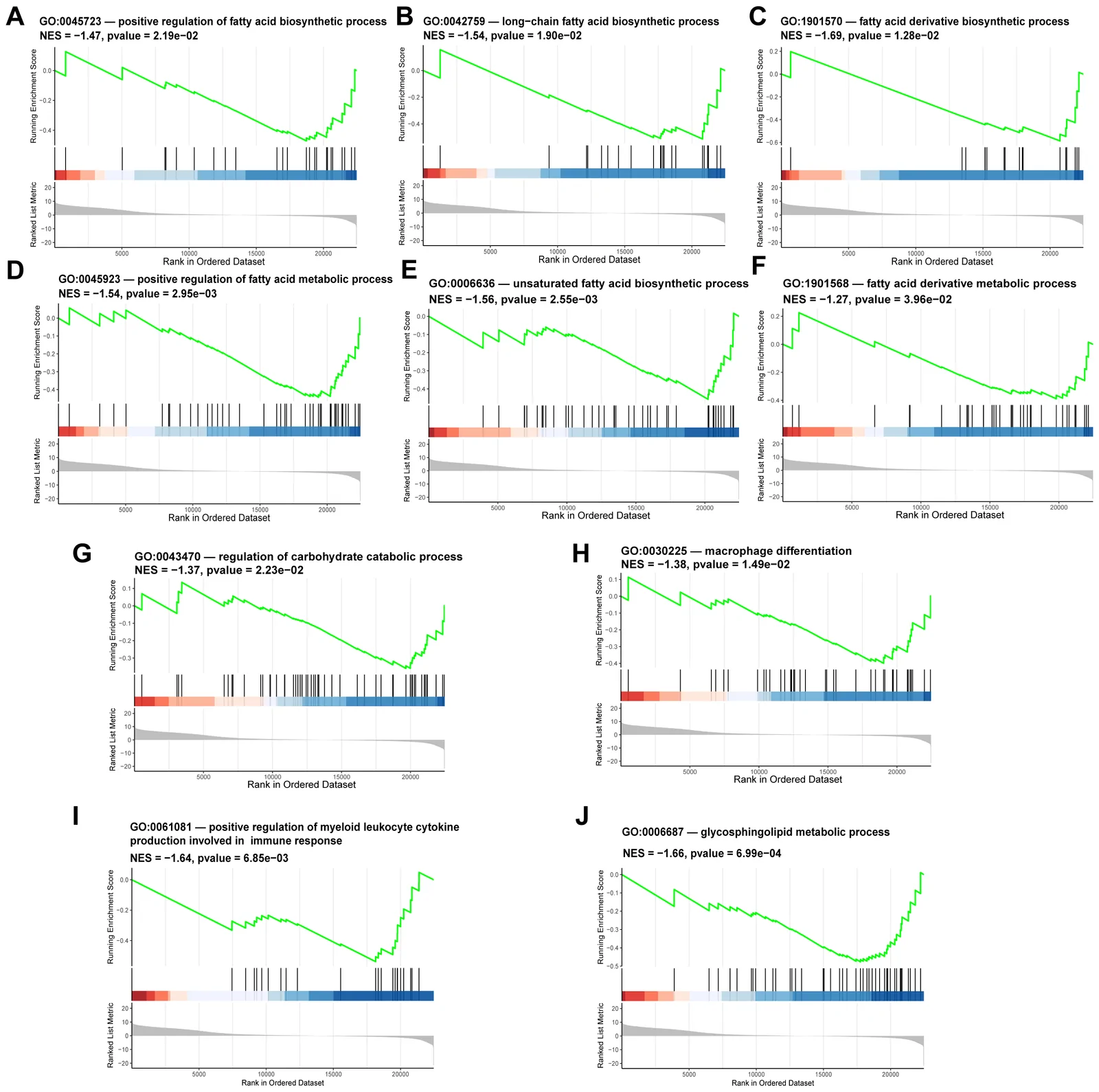
GSEA analyses. **(A–J)** Gene set enrichment analysis showing significant suppression in the model group (NES < −1, nominal p < 0.05) of the following GOBP pathways: **(A)** positive regulation of fatty-acid biosynthetic process; **(B)** long-chain fatty-acid biosynthetic process; **(C)** fatty-acid derivative biosynthetic process; **(D)** positive regulation of fatty-acid metabolic process; **(E)** unsaturated fatty-acid biosynthetic process; **(F)** fatty-acid derivative metabolic process; **(G)** regulation of carbohydrate catabolic process; **(H)** macrophage differentiation; **(I)** positive regulation of myeloid-leukocyte cytokine production involved in immune response; **(J)** glycosphingolipid metabolic process.

Differential expression analysis (model vs. normal) identified 2,532 DEGs meeting |log2FC| > 1 and FDR < 0.05, including 1,790 upregulated and 742 downregulated genes in the model group (**Figure 3A**); a DEG heatmap is shown in **Figure 3B**. GO and KEGG enrichment revealed overrepresentation of GOBP terms related to epithelial cell proliferation, positive regulation of cytokine production, and fatty-acid metabolic process (**Figure 3C**). GOCC terms were enriched for collagen-containing extracellular matrix, receptor complex, and membrane microdomain (**Figure 3D**), while GOMF terms included receptor ligand activity, G protein-coupled receptor binding, and phospholipid binding (**Figure 3E**). KEGG analysis further highlighted PPAR signaling, glycolysis/gluconeogenesis, and glutathione metabolism (**Figure 3F**). Consistently, GSEA showed significant negative enrichment of multiple lipid-metabolism-related pathways in wart-bearing mice, including positive regulation of fatty-acid biosynthetic process (NES = −1.473, P = 0.0219), long-chain fatty-acid biosynthetic process (NES = −1.536, P = 0.0190), fatty-acid derivative biosynthetic process (NES = −1.694, P = 0.0128), positive regulation of fatty-acid metabolic process (NES = −1.543, P = 0.0030), unsaturated fatty-acid biosynthetic process (NES = −1.560, P = 0.0025), fatty-acid derivative metabolic process (NES = −1.269, P = 0.0396), regulation of carbohydrate catabolic process (NES = −1.375, P = 0.0223) and glycosphingolipid metabolic process (NES = −1.662, P = 0.0007; **Figures 4A–G, J**). These results support the suppression of FAM-related programs in wart-bearing mice. In addition, immune-related terms were negatively enriched, including macrophage differentiation (NES = −1.376, P = 0.0149) and positive regulation of myeloid leukocyte cytokine production involved in immune response (NES = −1.638, P = 0.0068; **Figures 4H, I**), providing transcriptomic support for altered immune-related processes in the wart model.

### FAM-DEGs in cutaneous warts

**Figure 5:**
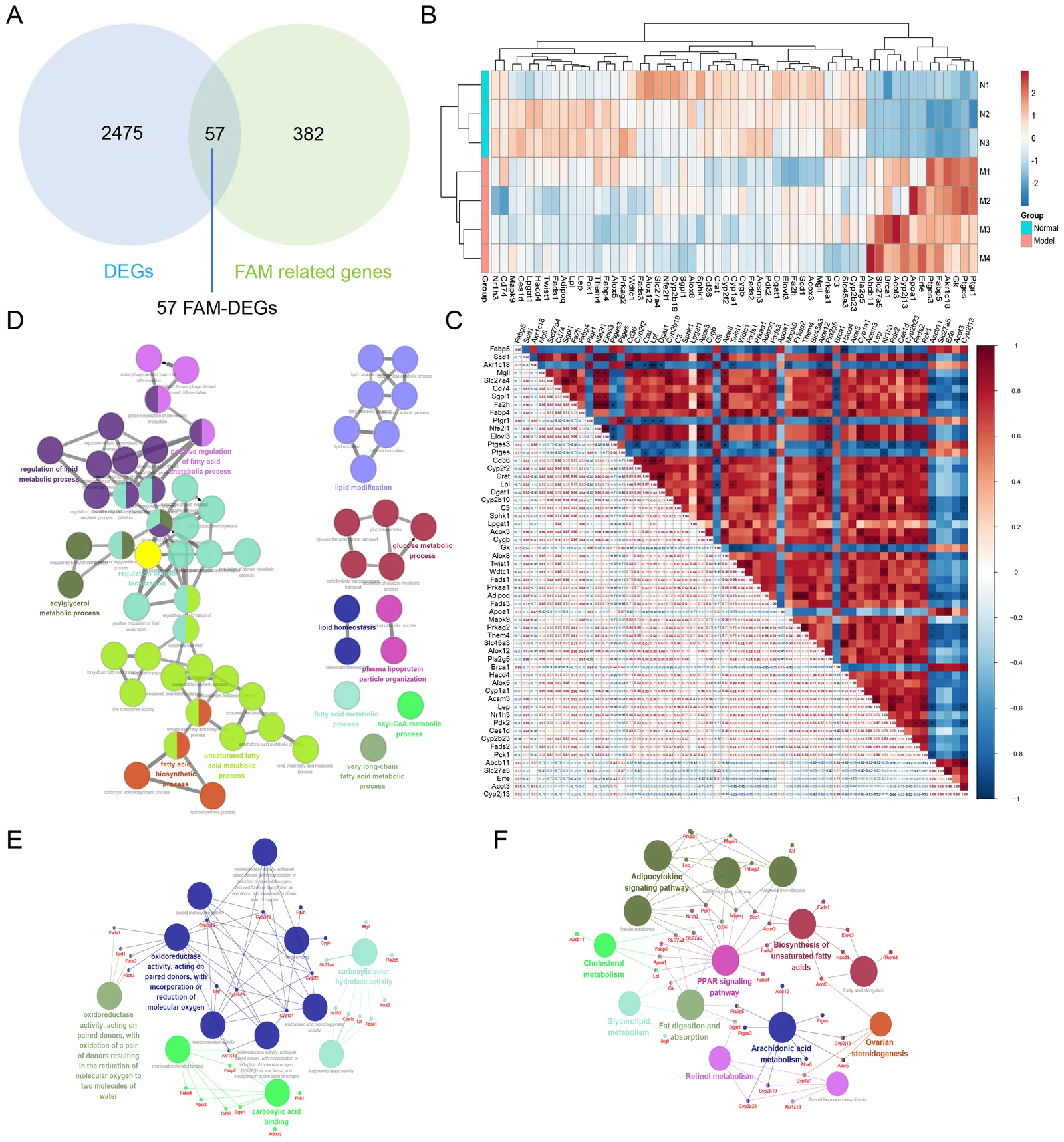
Analysis of FAM-DEGs. **(A)** Venn diagram showing the intersection between DEGs and the curated FAM gene set (GO:0006631 and BP offspring). **(B)** Heatmap of FAM-DEG expression across samples. **(C)** Pairwise Pearson correlation heatmap of FAM-DEGs. Numeric values indicate signed correlation coefficients (r); the color scale denotes direction and magnitude (red, positive; blue, negative); asterisks indicate two-sided P-value levels (*P < 0.05, ***P < 0.001). **(D-F)** Functional enrichment of FAM-DEGs: **(D)** GO Biological Process, **(E)** GO Molecular Function, and **(F)** KEGG pathway enrichment.

Given the central role of FAM in skin homeostasis, we examined FAM-related genes in cutaneous warts. A curated FAM gene set (n = 439) was constructed from fatty acid metabolic process (GO:0006631) and its BP offspring (GO.db) and mapped to mouse genes via org.Mm.eg.db for downstream analyses. Intersecting all DEGs with this set yielded 57 FAM-DEGs (**Figure 5A**), of which 13 were upregulated and 44 were downregulated in the model group versus controls (**Figure 5B**). Pairwise correlations among these 57 genes are shown as a heatmap (**Figure 5C**). For example, Scd1, Mgll, Slc27a4, and Sgpl1 correlated positively with Fa2h, Nfe2l1, Elovl3, Cyp2b19, and Acox3, whereas Slc27a4 and Sgpl1 were negatively correlated with Abcb11 and Cyp2j13.

Functional network analysis using ClueGO (Cytoscape) showed significant enrichment for GOBP terms including regulation of lipid metabolic process, glucose metabolic process, acylglycerol metabolic process, acyl-CoA metabolic process, and very-long-chain fatty-acid metabolic process (**Figure 5D**). At the molecular-function level, enriched terms included oxidoreductase activity (acting on paired donors, with incorporation or reduction of molecular oxygen), carboxylic acid binding, and carboxylic ester hydrolase activity (**Figure 5E**). KEGG pathways were enriched for adipocytokine signaling, PPAR signaling, biosynthesis of unsaturated fatty acids, arachidonic acid metabolism, and fat digestion and absorption (**Figure 5F**).

### Identification of hub FAM-DEGs and construction of the regulatory network

**Figure 6:**
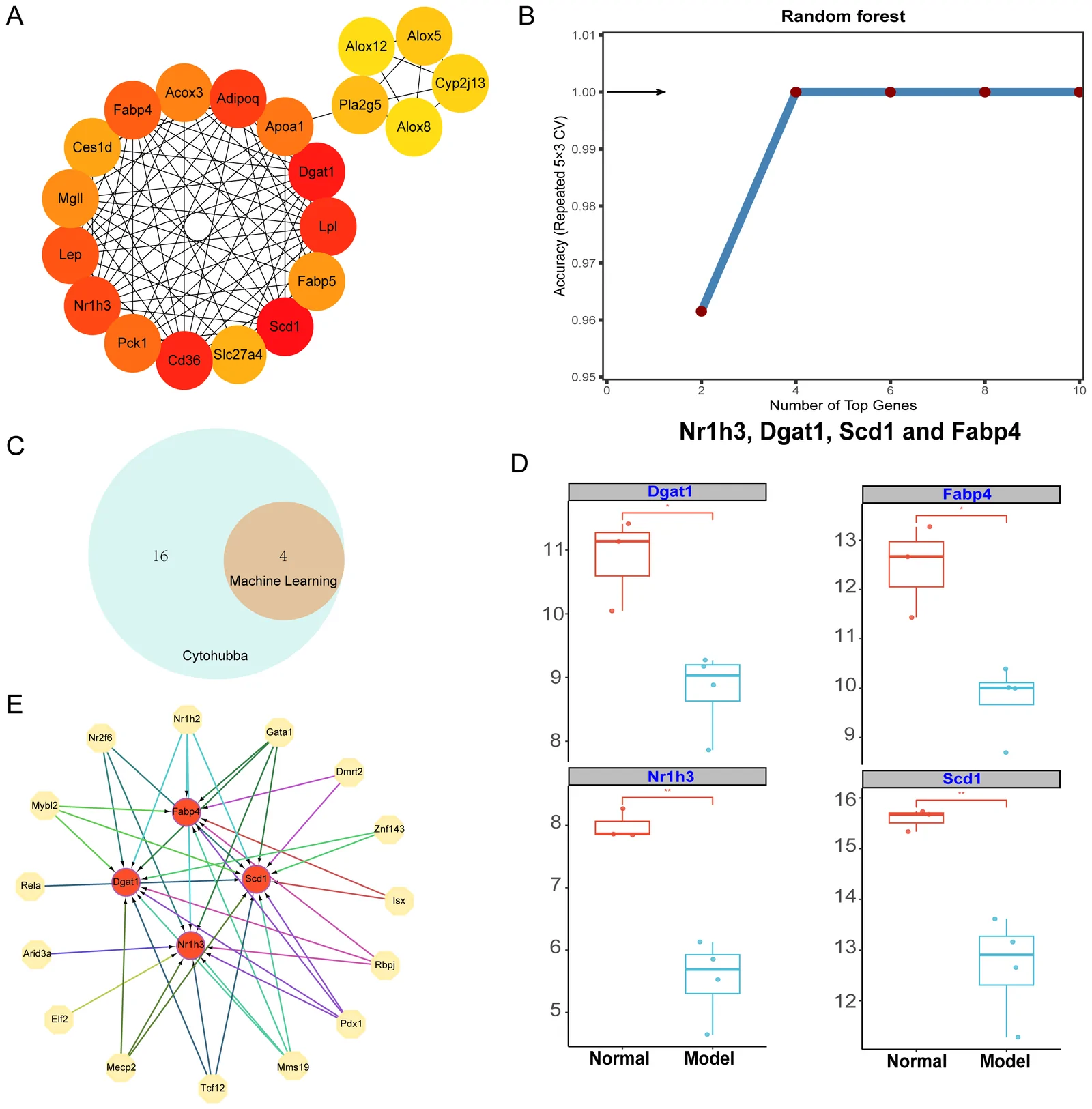
Identification of key FAM-DEGs. **(A)** MCC-based prioritization of candidate hub FAM-DEGs. **(B)** Random-forest selection of informative FAM-DEG features. **(C)** Venn diagram showing the intersection of candidate hubs, yielding four hub FAM-DEGs. **(D)** Boxplots of RNA-seq-derived expression values for the four hub FAM-DEGs in model and normal skin, not qPCR validation data. **(E)** Predicted transcription-factor regulatory network for the four hub FAM-DEGs.

PPI network analysis of the 57 FAM-DEGs was performed using STRING (https://string-db.org/); the network is shown in **Supplementary Figure 2**. To prioritize hubs, we applied the Maximal Clique Centrality (MCC) algorithm in cytoHubba (Cytoscape) alongside machine-learning–based screening. MCC identified 20 candidate hub genes: Fabp5, Pck1, Adipoq, Nr1h3, Alox12, Cyp2j13, Fabp4, Acox3, Pla2g5, Mgll, Lep, Lpl, Apoa1, Alox5, Cd36, Slc27a4, Ces1d, Scd1, Dgat1, and Alox8 (**Figure 6A**). Random-forest analysis further prioritized Nr1h3, Dgat1, Scd1, and Fabp4 as the most informative features among these candidates (**Figure 6B**).

Intersecting the MCC-derived candidates with the machine-learning selection yielded Nr1h3, Dgat1, Scd1, and Fabp4 as core FAM-DEGs (**Figure 6C**; full gene names in **Table 2**). All four were significantly downregulated in wart tissues compared with controls based on RNA-seq-derived expression values rather than qPCR measurements (**Figure 6D**). To delineate upstream regulation, we used iRegulon (Cytoscape) to predict transcription factors (TFs), retaining the top 15 by normalized enrichment score: Nr1h2, Nr2f6, Gata1, Dmrt2, Mybl2, Znf143, Isx, Rbpj, Pdx1, Mms19, Tcf12, Mecp2, Elf2, Arid3a, and Rela (**Figure 6E**). The TF-hub network indicates that Nr1h2, Gata1, Pdx1, and Mms19 are predicted to co-regulate all four hubs; Nr2f6 and Rbpj are predicted to regulate Nr1h3, Dgat1, and Fabp4; Mecp2 and Tcf12 are predicted to regulate Nr1h3, Dgat1, and Scd1; and Mybl2 is predicted to regulate Dgat1, Scd1, and Fabp4.

**Table 2:** Hub FAM-DEG.

Gene	Full name
*Nr1h3*	Nuclear receptor subfamily 1 group H member 3
*Dgat1*	Diacylglycerol O-acyltransferase 1
*Scd1*	Stearoyl-CoA desaturase 1(Δ9-desaturase)
*Fabp4*	Fatty acid binding protein 4

### Links between hub FAM-DEGs and immune infiltrates in cutaneous warts

**Figure 7:**
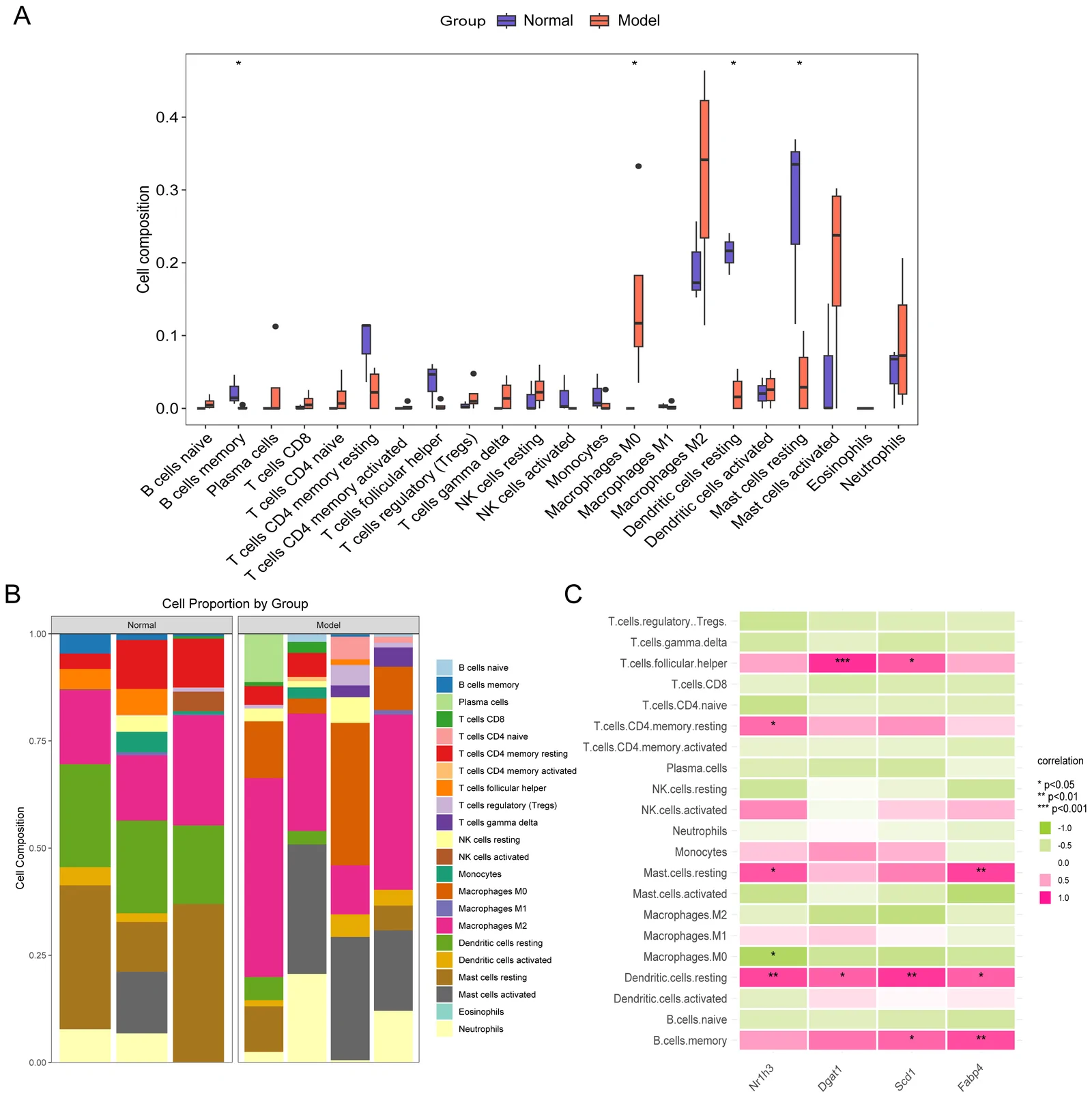
Associations between key FAM-DEGs and immune-cell infiltration. **(A)** Inferred immune-cell composition by group. **(B)** Inferred immune-cell composition by individual sample. **(C)** Correlations between hub FAM-DEGs and immune-cell subsets. *P < 0.05, **P < 0.01, ***P < 0.001.

Immune-cell infiltration was inferred using CIBERSORT with the LM22 leukocyte signature, estimating relative abundances of 22 cell types. Four cell types differed significantly between model and normal groups (**Figure 7A**). The model group exhibited a higher proportion of M0 macrophages, while memory B cells, resting dendritic cells, and resting mast cells were more abundant in the normal group (**Figure 7B**). Correlating hub FAM-DEG expression with inferred immune-cell proportions revealed significant associations (**Figure 7C**): Nr1h3 was negatively correlated with M0 macrophages and positively correlated with resting memory CD4**^+^** T cells, resting mast cells, and resting dendritic cells; Dgat1 correlated positively with T follicular helper cells and resting dendritic cells; Scd1 correlated positively with T follicular helper cells, resting dendritic cells, and memory B cells; and Fabp4 correlated positively with resting mast cells, resting dendritic cells, and memory B cells.

### HPV16 E6/E7 expression suppresses fatty acid metabolism-related genes in keratinocytes

**Figure 8:**
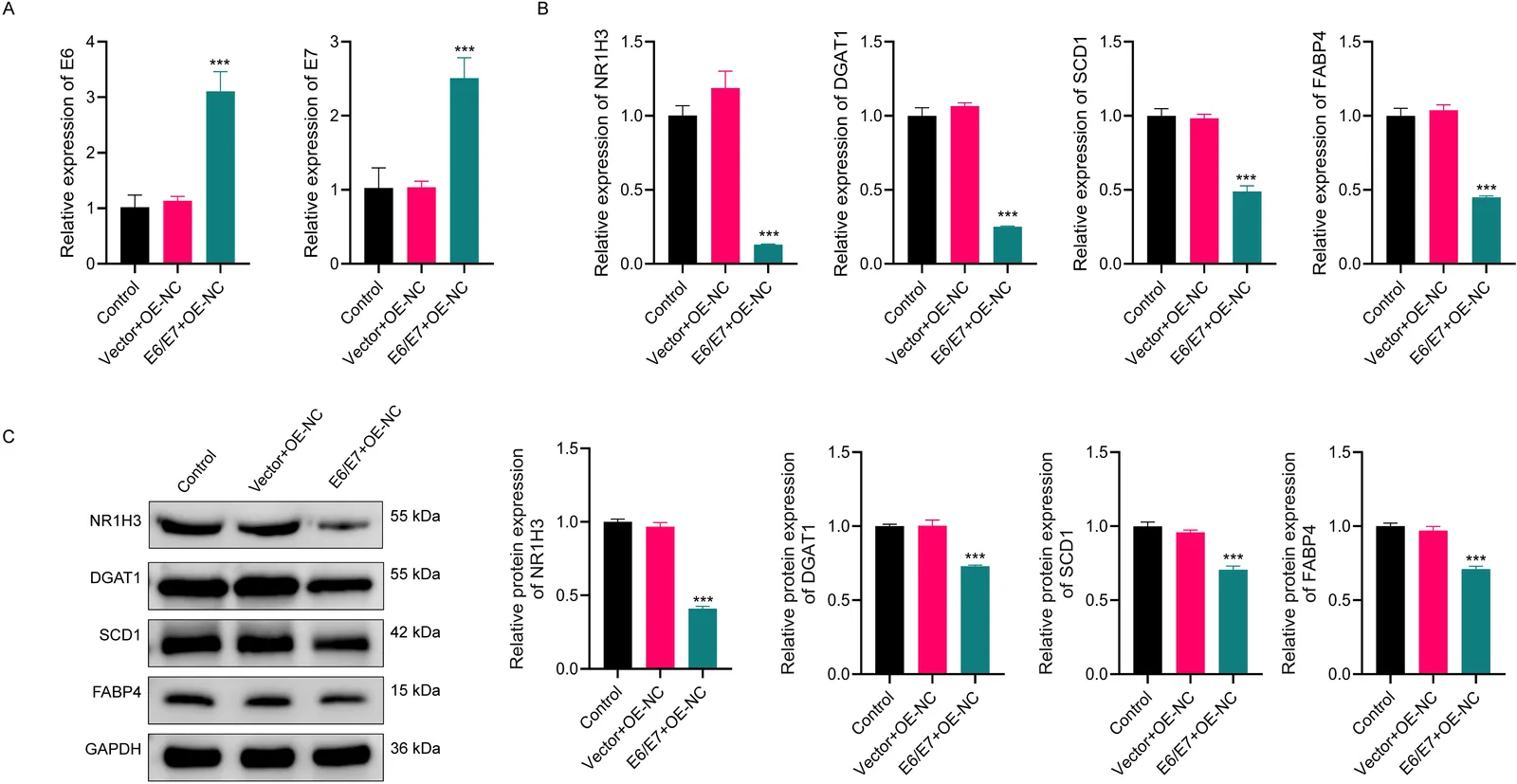
HPV16 E6/E7 expression downregulates FAM-hub genes in HaCaT keratinocytes. **(A, B)** qRT-PCR analysis showing mRNA expression of E6/E7 **(A)** and NR1H3, DGAT1, SCD1, and FABP4 **(B)** in HaCaT cells; OE-NC denotes empty/negative-control overexpression vector. **(C)** Representative western blot images and quantification of protein expression levels of NR1H3, DGAT1, SCD1, and FABP4. ***P<0.001.

To validate the transcriptomic findings in keratinocytes, we transiently co-transfected HaCaT cells with HPV16 E6/E7 expression plasmids to simulate early viral-gene expression. Quantitative RT-PCR confirmed successful E6/E7 transfection in the E6/E7 + OE-NC group, with E6 and E7 mRNA levels markedly higher than in the control or vector-only groups (**Figure 8A**). The minimal amplification signals detected in control samples were near the limit of detection and represented assay background rather than endogenous or contaminating E6/E7 expression. Consistent with the *in vivo* RNA-seq data, E6/E7 expression significantly reduced the mRNA levels of NR1H3, DGAT1, SCD1, and FABP4 (**Figure 8B**).

Western blot analysis further indicated that compared with the control group, the protein levels of NR1H3, DGAT1, SCD1, and FABP4 in cells with E6/E7 expression were significantly decreased (**Figure 8C**). The magnitude of mRNA and protein reduction was not identical across targets, which may reflect differences in protein half-life, translation efficiency, post-translational regulation, and assay dynamic range. These results support the conclusion that HPV16 E6/E7 expression is sufficient to directionally suppress NR1H3 and key fatty acid metabolism-related genes in human keratinocytes, recapitulating part of the molecular pattern observed in papillomavirus-induced lesions *in vivo*.

### NR1H3 overexpression rescues E6/E7-induced proliferative and differentiation abnormalities in keratinocytes

**Figure 9:**
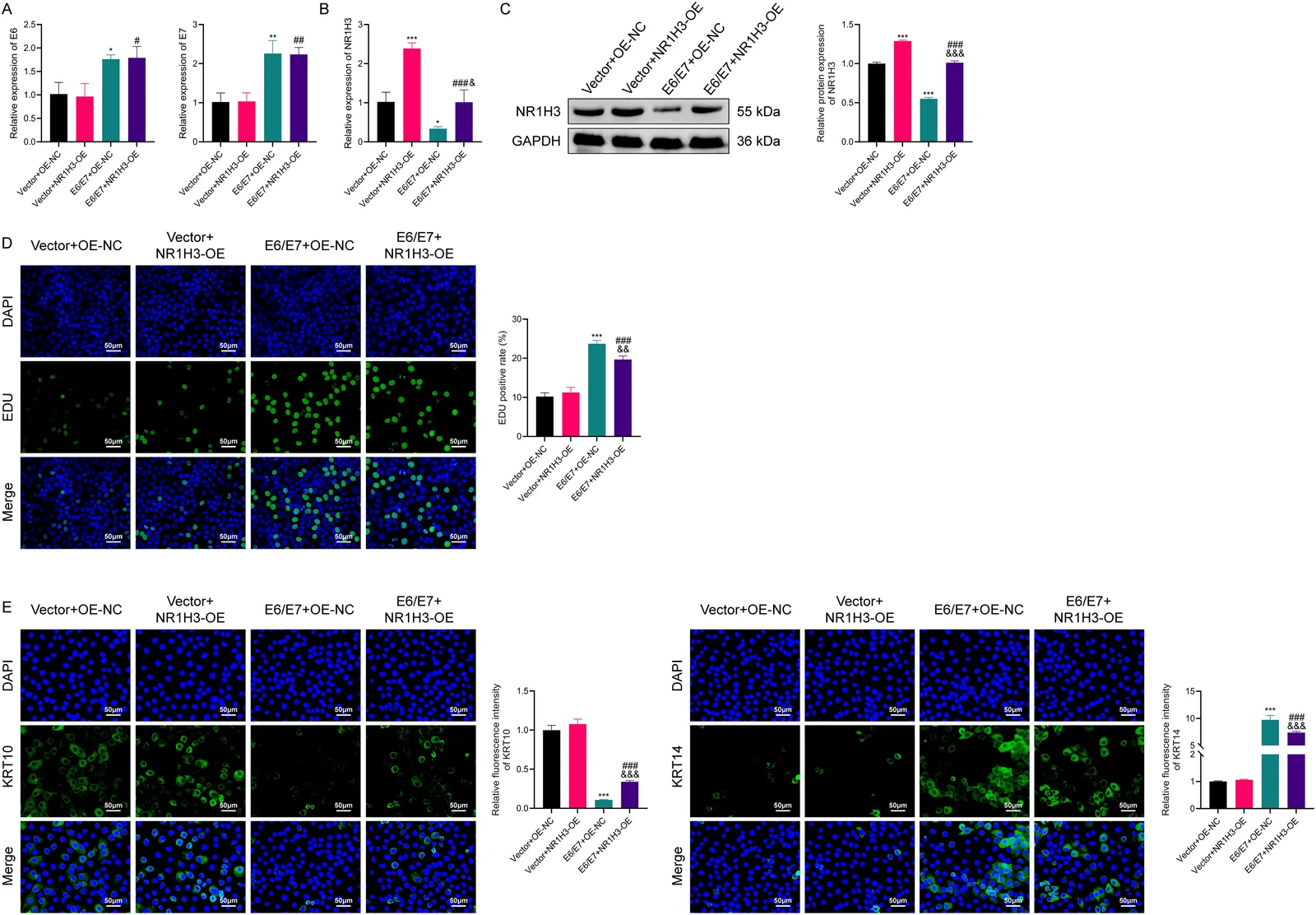
NR1H3 overexpression rescues E6/E7−induced phenotypic alterations in HaCaT cells. **(A, B)** qRT-PCR verified the mRNA expression levels of E6/E7 **(A)** and NR1H3 **(B)** among different groups. **(C)** Western blot analysis of NR1H3. **(D)** EdU proliferation experiment. EdU (green), DAPI nuclear counterstaining (blue). **(E)** Immunofluorescence staining of differentiation markers KRT10 (green) and KRT14 (green); cell nuclei stained with DAPI (blue). Scale bar: 50 μm. *P < 0.05, **P < 0.01, ***P < 0.001 vs. Vector+OE−NC group. #P < 0.05, ##P < 0.01, ###P < 0.001 vs. Vector+NR1H3-OE group. &P < 0.05, &&P < 0.01, &&&P < 0.001 vs. E6/E7+OE-NC group.

We selected NR1H3 for in-depth rescue experiments because it encodes LXRalpha, a ligand-responsive nuclear receptor that integrates lipid homeostasis, inflammatory regulation, and epithelial differentiation, and because it was both downregulated in the MmuPV1 RNA-seq dataset and suppressed by HPV16 E6/E7 in HaCaT cells. To determine whether NR1H3 contributes to the biological effects of HPV E6/E7, NR1H3 was overexpressed in HaCaT cells in the presence or absence of E6/E7. qRT-PCR and western blot analyses confirmed effective NR1H3 overexpression and showed that E6/E7-induced suppression of NR1H3 protein was significantly reversed by NR1H3 overexpression (**Figures 9A–C**).

Functional assays revealed that E6/E7 expression markedly enhanced keratinocyte proliferation, as evidenced by increased EdU incorporation, whereas NR1H3 overexpression significantly attenuated this proliferative response (**Figure 9D**). Immunofluorescence staining further demonstrated that E6/E7 disrupted keratinocyte differentiation, characterized by increased expression of the basal marker KRT14 and decreased expression of the differentiation marker KRT10. Importantly, NR1H3 overexpression partially restored the differentiation balance, reducing KRT14 expression while increasing KRT10 levels compared with the E6/E7 + OE-NC group (**Figure 9E**).

These findings suggest that NR1H3 may contribute to the regulation of keratinocyte proliferation and differentiation under HPV oncogene–driven conditions.

### NR1H3 modulates lipid accumulation and inflammatory signaling through the ABCA1/NF-κB pathway

**Figure 10:**
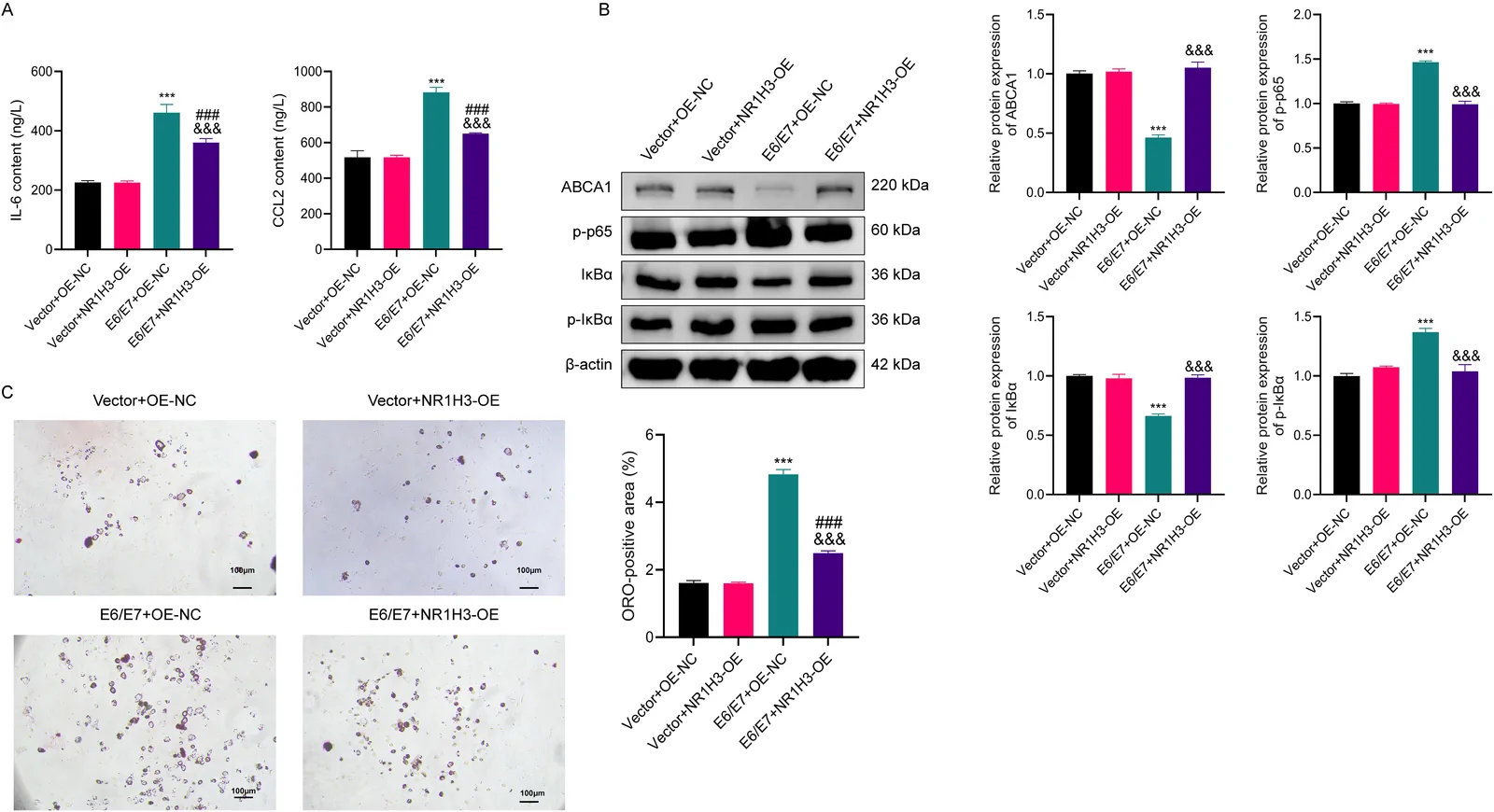
Analysis of inflammatory cytokines and signaling pathway regulation after NR1H3 rescue. **(A)** ELISA detecting the levels of IL-6 and CCL2. **(B)** Western blot analysis of ABCA1, p-p65, IkBalpha, and p-IkBalpha protein expression. **(C)** Oil Red O staining and image-based quantification of lipid-droplet accumulation; representative fields are shown with enlarged display for visual assessment. Graphical summary: Papillomavirus infection is associated with fatty-acid metabolic remodeling and NR1H3/ABCA1-related inflammatory signaling in cutaneous wart formation. ***P < 0.001 vs. Vector+OE‑NC group. ###P < 0.001 vs. Vector+NR1H3-OE group. &&&P < 0.001 vs. E6/E7+OE-NC group.

Given the established role of NR1H3/LXRalpha in lipid homeostasis, we next evaluated lipid accumulation and inflammatory signaling. ABCA1 was examined because it is a canonical NR1H3/LXR target involved in cholesterol efflux and membrane lipid homeostasis, providing a plausible link between NR1H3 activity and inflammatory NF-kB signaling. ELISA indicated that E6/E7 expression significantly increased the secretion of pro-inflammatory cytokines IL-6 and CCL2, while NR1H3 overexpression significantly attenuated this inflammatory response (**Figure 10A**). At the molecular level, western blot analysis revealed that E6/E7 expression inhibited ABCA1 protein expression and activated the NF-kB pathway, as shown by increased phosphorylation of p65 and IkBalpha and decreased total IkBalpha expression (**Figure 10B**). In contrast, NR1H3 overexpression restored ABCA1 expression, inhibited NF-kB activation, reduced p-p65 and p-IkBalpha levels, and stabilized IkBalpha. Oil Red O staining showed that E6/E7 expression significantly increased lipid-droplet accumulation in HaCaT cells, while NR1H3 overexpression significantly reduced lipid deposition (**Figure 10C**).

Overall, these results suggest that NR1H3 modulates HPV-induced lipid accumulation and inflammatory signaling, potentially through regulation of the ABCA1/NF-κB axis.

## Discussion

In the general population, the prevalence of cutaneous warts can be as high as 33%, whereas in immunocompromised individuals prevalence and even incidence may reach up to 90% (Kombe Kombe et al., 2020). Epidemiologic studies further indicate that a history of HPV infection increases the risk of cutaneous neoplasms (Chen et al., 2021). The molecular mechanisms underlying cutaneous warts remain incompletely defined, and effective treatments for recurrent, recalcitrant lesions are limited and warrant further investigation (Leerunyakul et al., 2022). In recent years, MmuPV1 infection of murine tail skin has provided an *in vivo* surrogate model in which the virus replicates within stratified squamous epithelium and forms papillomatous lesions, enabling deeper investigation of cutaneous papillomavirus infection (Best et al., 2020). Here, we asked two central questions: first, whether papillomavirus-induced cutaneous wart formation is associated with coordinated suppression of FAM-related programs; and second, whether viral early-gene activity directly downregulates candidate FAM regulators in keratinocytes and whether restoring one such regulator (NR1H3) can attenuate HPV-driven cellular abnormalities. GSEA likewise demonstrated that lipid metabolic pathways were significantly downregulated in lesions relative to controls. To our knowledge, FAM-related DEGs in cutaneous papillomavirus disease have not been systematically characterized previously. These findings support a metabolic component of wart biology and identify candidate regulatory nodes for future functional testing, but they do not by themselves prove that these genes are sufficient therapeutic targets *in vivo*.

Our transcriptome analysis identified four core downregulated FAM-related genes in the skin warts of mice infected with MmuPV1: Nr1h3, Dgat1, Scd1 and Fabp4. In human psoriasis, LXRα (Nr1h3) is downregulated, with greater suppression in severe disease, impairing epidermal function (Mehta et al., 2013). Because LXRα senses oxysterols to coordinate cholesterol and FAM (Fu et al., 2001), reduced Nr1h3 in warts likely reflects attenuated LXR signaling. This is consistent with our GSEA showing negative enrichment of fatty-acid pathways, supporting a model in which diminished LXR transactivation contributes to metabolic reprogramming in wart tissue. Dgat1 catalyzes the terminal step of triacylglycerol synthesis by esterifying diacylglycerol with fatty acyl-CoA, diverting fatty acids from metabolically active pools into lipid droplets and chylomicrons for storage and transport (van Rijn et al., 2018). Beyond adipose biology, Dgat1 channels fatty acids into neutral lipids essential for cutaneous homeostasis. In mice, Dgat1−/− causes sebaceous-gland atrophy, loss of type II wax diesters, dry fur, impaired water repellency, and hypothermia. In our wart tissues, Dgat1 was downregulated and fatty-acid pathways were negatively enriched, consistent with reduced triacylglycerol synthesis and disturbed sebaceous/epidermal lipid composition that may weaken barrier function and reshape the local immune milieu (Chen et al., 2002). Scd1 is the rate-limiting Δ9-desaturase for monounsaturated fatty-acid (MUFA) biosynthesis and a key determinant of cutaneous lipid homeostasis. In our wart tissues, Scd1 was downregulated, and fatty-acid pathways were negatively enriched. This suggests reduced MUFA availability, which may impair membrane fluidity, lipid-raft organization, sebaceous/epidermal lipid composition, and barrier integrity. Prior studies link Scd1-MUFA metabolism to innate immune signaling via context-dependent modulation of the cGAS–STING–TBK1 axis (Geng et al., 2024). In mouse models, loss of the Scd1 gene disrupts sebum composition and compromises epidermal barrier function (Sampath and Ntambi, 2014), it also perturbs sebaceous/epidermal lipid homeostasis and reshapes the skin microbiome (Pyle et al., 2023). Collectively, these alterations suggest that Scd1 deficiency may increase the skin’s susceptibility to persistent viral infection. Together, these observations support a model in which diminished Scd1 activity contributes to metabolic and immune dysregulation in papillomatous skin. Fabp4 encodes a fatty-acid binding protein that can associate with adenosine kinase to form a hormone-like functional complex, thereby regulating intra- and extracellular ATP levels (Prentice et al., 2021). Independent studies in irradiated skin indicate that adipocyte-derived lipids and Fabp4 enhance cutaneous stress resilience and DNA-damage repair, consistent with our observation that fatty-acid programs are disrupted in wart tissue (Xiao et al., 2020). Notably, Pan et al. (2017) reported that Fabp4 knockout mice exhibit diminished protection against cutaneous viral infection. Taken together, these data support the hypothesis that reduced Fabp4 in our MmuPV1 model may compromise DNA-repair capacity and antiviral defense.

Among the four core genes, we chose NR1H3 for in-depth mechanism exploration based on its role as a ligand-responsive transcriptional regulator that links lipid metabolism, inflammatory responses, and cell differentiation. NR1H3/LXRalpha controls lipid efflux programs including ABCA1 and can antagonize inflammatory transcriptional pathways, thereby providing a mechanistic rationale for examining the ABCA1/NF-kB axis (Venkateswaran et al., 2000; Joseph et al., 2003). Using an *in vitro* keratinocyte model expressing HPV16 E6/E7, we found that E6/E7 was sufficient to inhibit NR1H3 at the mRNA and protein levels, accompanied by downregulation of key lipid metabolism effectors. Functionally, this inhibitory effect was associated with enhanced keratinocyte proliferation, impaired differentiation, lipid-droplet accumulation, and activation of inflammatory signals. Importantly, NR1H3 overexpression partially restored these phenotypes. At the signaling level, ABCA1 downregulation in E6/E7-expressing keratinocytes was accompanied by NF-kB activation, increased phosphorylation of p65 and IkBalpha, and increased secretion of IL-6 and CCL2. Restoring NR1H3 expression reversed these changes, supporting a model in which papillomavirus early-gene activity suppresses the NR1H3/ABCA1 pathway and thereby promotes lipid accumulation and NF-kB-driven inflammatory signaling. These *in vitro* data suggest a hypothesis that restoring NR1H3/LXRα activity might attenuate HPV-associated keratinocyte dysfunction. However, this hypothesis requires direct *in vivo* testing in the MmuPV1 model using LXR agonists (e.g., T0901317, GW3965) or keratinocyte-specific NR1H3 knockout before any therapeutic claim can be made. Because HPV16 E6/E7 are central oncogenic drivers in HPV-associated malignancies, the observation that NR1H3 overexpression attenuated E6/E7-induced proliferative and inflammatory phenotypes may also have implications for lipid-metabolic vulnerabilities in HPV16-positive cancers; however, cancer models will be required to test this possibility directly (Doorbar et al., 2015).

Our immune infiltration analysis revealed remodeling of the local immune environment in HPV-infected skin. The proportion of M0 macrophages increased in lesion tissue, while memory B cells, resting dendritic cells, and resting mast cells decreased. These CIBERSORT-based findings should be interpreted as computational estimates from bulk RNA-seq rather than direct histologic quantification. Nevertheless, the direction of the inferred immune changes is consistent with enrichment results for immune-response and cytokine-related pathways and with H&E evidence of papillomatous epithelial remodeling. Correlation analysis showed that FAM hub genes such as NR1H3 were positively correlated with resting immune-cell subsets and negatively correlated with M0 macrophages, suggesting that local lipid-metabolic disturbance may be linked to immune-microenvironment remodeling. Future immunohistochemistry or flow-cytometry studies will be needed to validate the inferred immune-cell changes directly.

Although the present study focused on benign cutaneous warts rather than HPV-associated malignancies, our finding that NR1H3 overexpression attenuated E6/E7-induced proliferation and inflammatory phenotypes may have broader implications for HPV16-positive cancers. HPV16 E6/E7 are central drivers in cervical, head and neck, and other anogenital cancers, where lipid metabolic reprogramming is increasingly recognized as a hallmark. The NR1H3/ABCA1/NF-κB axis identified here could represent a metabolic vulnerability in HPV16-driven malignancies, warranting investigation in cancer-specific models. However, direct extrapolation from benign keratinocytes to malignant contexts would be premature without appropriate cancer models.

This study has some limitations, which need to be acknowledged. Although our *in vitro* rescue experiments provided mechanistic support for the pathway centered on NR1H3, further *in vivo* gene manipulation, pharmacologic activation with NR1H3/LXR agonists, or functional loss studies in the MmuPV1 model are needed to clarify causal relationships and therapeutic potential. Second, HPV16 E6/E7 expression represents an experimental model of viral early-gene activity and may not fully reflect the complexity of papillomavirus-host interactions *in vivo*. Third, inoculated mice that did not develop visible warts were not included in the RNA-seq comparison; future studies should analyze wart-negative inoculated animals to distinguish exposure-related from lesion-specific transcriptional changes. Fourth, the present study did not include direct viral integration analysis or additional molecular assays of productive infection in each tissue sample. Fifth, lipid-droplet accumulation was quantified by image-based analysis of Oil Red O-stained areas rather than by spectrophotometric extraction of the dye. Although image-based quantification is widely used and correlates well with lipid content, spectrophotometric measurement would provide a more directly quantitative readout. Future studies should include both approaches to enhance quantitative rigor. Finally, this study mainly focused on epidermal keratinocyte-level changes, and the roles of dermal components such as fibroblasts and adipocytes in metabolic reprogramming deserve further exploration.

## Conclusion

In conclusion, papillomavirus infection is associated with marked suppression of fatty-acid-metabolism-related pathways in cutaneous lesions. Integrated transcriptomic and functional analyses identify NR1H3, Dgat1, Scd1, and Fabp4 as candidate regulatory genes linked to keratinocyte dysregulation and immune-microenvironment remodeling. These findings suggest that programNR1H3-centered metabolic regulation may be associated with papillomavirus-related lipid accumulation and inflammatory signaling, while definitive causal and therapeutic validation requires further *in vivo* studies.

## Data Availability

The datasets presented in this study can be found in online repositories. The names of the repository/repositories and accession number(s) can be found in the article/**Supplementary Material**.

## Supplementary material

Supplementary Figure 1 MmuPV1 DNA loads in mouse tail tissues from each group were determined by qPCR.

Supplementary Figure 2 PPI network of the 57 FAM-DEGs. The network was constructed using the STRING database and visualized in Cytoscape. Each node represents a protein encoded by a differentially expressed gene related to FAM-DEG. Edges indicate functional and physical protein associations.
